# “It All Comes Down to the Teens”: An Actor-Partner-Interdependence Model of Trust, Engagement and Satisfaction With the School in Parent-Adolescent Dyads

**DOI:** 10.5964/ejop.15335

**Published:** 2025-11-28

**Authors:** Petru Lucian Curșeu, Arcadius Florin Muntean, Mihai Tucaliuc

**Affiliations:** 1 Department of Psychology, Babeş – Bolyai University Cluj-Napoca, Romania; 2 Department of Organization, Open Universiteit, Heerlen, the Netherlands; Università Cattolica del Sacro Cuore, Milan, Italy

**Keywords:** trust, parental school engagement, satisfaction with the school, adolescents, parent adolescent dyads, student school engagement

## Abstract

**Objectives:**

Our study underscores the relevance of focusing on parent-adolescent dyads in order to understand the interplay between school trust, engagement and satisfaction with the school by testing a model in which school engagement mediates the association between trust in the school and satisfaction with the school.

**Methods:**

We use an actor-partner-interdependence-model to test the mediating role of school engagement between trust and satisfaction with the school in 506 parent-adolescent dyads in Romania.

**Results:**

The results show that when these variables are jointly evaluated in parent-adolescent dyads, only student engagement is a significant dyadic (couple) mediator, such that it is positively predicted both by student and parental trust in the school and predicts both student and parental satisfaction with the school. Our results also reveal a partner only effect on parental school engagement that is positively predicted by student trust in the school. Moreover, our results also reveal that parental trust in the school has a positive association with parental as well as student satisfaction with the school. An emergent result is that female students report a higher engagement and satisfaction with the school than male students do.

**Conclusions:**

In line with our results, we urge school administrators to provide resources and create platforms for a joint involvement of parents and adolescents in relevant school-related activities.

Both parental and adolescent engagement with the school are essential drivers of academic performance and wellbeing at school (Goodall & Montgomery, 2014; Lei et al., 2018; Mielityinen et al., 2023) and literature to date has extensively explored factors that drive school engagement. Student school engagement reflects the effort and energy invested by students in school-related activities and factors like student self-efficacy, motivation, personality as well as classroom climate and the quality of relationships with the teachers have been studied as key antecedents (Fredricks et al., 2004; Lai et al., 2021; Mielityinen et al., 2023). Parental school engagement refers to parents’ involvement in school-related activities in order to build a sense of joint ownership and support (together with the school) children’s learning journeys. Among the antecedents of parental school engagement, trust in the school and the quality of the relationship with the school and teachers were the key antecedents explored so far (Adams et al., 2022; Goodall & Montgomery, 2014; Romero, 2015).

As parental and student school engagement reflect behavioral intentions, factors that pertain to the children-parent relation are likely to impact on school engagement as well as on its antecedents and consequences. To date however, studies mostly focused on personal and classroom related antecedents and did not extensively explore the way in which the dyadic relations between adolescents and their parents impact on student and parental school engagement and on the interplay of engagement with other school attitudes. We use the Actor-Partner-Interdependence-Model (APIM see Cook & Kenny, 2005; Kenny, 1996, 2018) to explore the interplay between trust in the school, engagement and satisfaction with the school in parent–adolescent dyads. APIM is a suitable modeling approach that captures the interdependence between attitudes and behavioral intentions in interacting dyads and as such it was extensively used in studying close relationships, including family dynamics (Cook & Kenny, 2005; Michael & Hasida, 2024; Ledermann et al., 2011). In our study, the APIM analytic approach allowed us to simultaneously test the relationships between trust, engagement and satisfaction with the school evaluated by adolescents and their parents, therefore it captures these associations as shaped by the relationship dynamics in adolescent–parent dyads.

To summarize, our study extends the literature on school engagement that chiefly focused on student, school and classroom level antecedents of student and parental engagement and we build on the APIM to test, in parent–adolescent dyads, the mediating role of student and parent school engagement in the relation between trust in the school and satisfaction with the school. In doing so, our paper presents one of the first empirical attempts to jointly explore the interdependence of school attitudes in parent–adolescent dyads.

## Theoretical Background and Hypotheses

School engagement reflects the extent to which parents and the students are involved in school-related activities and practices such that a sense of commitment and ownership develops in relation to schools and the educational activities unfolding within (Goodall & Montgomery, 2014). School engagement has therefore a clear behavioral and action oriented focus. Given such clear behavioral elements specific to school engagement, we set out to test a model in which trust in the school (as a general positive evaluative orientation towards the school) predicts school engagement. A core tenet of the Social Quality Theory (SQT–Ward & Meyer, 2009) is that trust is at the very core of the social fabric of society as it fosters social inclusion, the development of social empowerment, socio-economic security as well as social cohesion. We elaborate on the SQT to argue that trust in the school reflects a set of generic positive evaluations, shared in parent–adolescent dyads, related to school’s mastery to convey and effectively transfer knowledge to students, legitimizing their educational authority to support students’ personal development and growth (Bormann et al., 2021; Niedlich et al., 2021). Trust in the school, as a general positive evaluation, fosters the legitimacy ascribed to the educational context represented by the school and is one of the key predictors of how students and parents interact and engage with the school.

Trust in education is a multifaceted construct that includes generalized trust in social institutions (education, political parties, public institutions) as well as emergent social trust that captures the relational dynamics among social actors (individuals, groups, organizations) (Bormann et al., 2021; Niedlich et al., 2021). Generalized trust reflects a generalized credibility of social institutions and organizations derived from a set of socio-cultural factors and individual evaluative tendencies (Niedlich et al., 2021), while in social relationships terms, trust refers to the willingness to accept vulnerability in relation to another social actor (group or individual) (Ward & Meyer, 2009), and it is typically the result of high quality communication among the partners. We build on the SQT notion that trust is a key antecedent of social engagement and social cohesion (Ward & Meyer, 2009) to explore the extent to which parents and students with a high trust in the school, are more likely to engage in school-related activities as compared to parents and students with a low level of trust in the school. We argue that evaluative tendencies concerning schools and the educational system in general are shared in adolescent–parent dyads, therefore evaluations of trust in the school tend to be interdependent in such dyads.

Whether trust refers to generic evaluative tendencies that refer to educational institutions in general, or trust in the school that emerges from good bidirectional communication between the school and the parents/students, we argue that trust reflects an effective and harmonious partnership in the educational process (Adams et al., 2022; Bormann et al., 2021; Houri et al., 2019; Romero, 2015). Partners engaged in harmonious social exchanges based on trust tend to display higher levels of engagement in the relationship. In support of these arguments, meta-analytic evidence shows that interventions aimed at increasing social support and effective feedback in communication have a significant positive effect on engagement (Knight et al., 2017). For the higher education context, studies have shown that students’ trust is an important predictor for their engagement with the school (Jones & Nangah, 2021), arguments fully supported by meta-analytic results (Roorda et al., 2017; Tao et al., 2022).

To summarize, in line with the SQT (Ward & Meyer, 2009), the social exchange perspective on social relations (Blau, 1964) and the cyclical model of trust in education (Niedlich et al., 2021), we argue that trust in the school reflects a set of positive generic evaluative tendencies shared in parent–adolescent dyads and is an antecedent for parental as well as adolescent school engagement. Most of the empirical evidence, however, is non-experimental, therefore the causal link between trust and engagement did not receive substantial evidence. An exception is a randomized control trial experiment reported in Houri et al. (2019) in which the authors have manipulated parental trust in school by involving the experimental parent group in an intense (daily exchanges), two way communication with the teachers, which ultimately increased their trust in the school. This trust manipulation eventually increased parental behavioral as well as relational school engagement (Houri et al., 2019). In line with these arguments, related to the causal association between trust in the school and engagement with the school, therefore we hypothesize that:

**Hypothesis 1**: Trust in the school has a positive association with school engagement.

Meta-analytic evidence supports a positive and significant association between employee engagement and satisfaction (Harter et al., 2002) as well as a positive association between student school engagement and academic performance and wellbeing in educational settings (Lei et al., 2018). Moreover, parental school engagement is an essential component of parental engagement with the overall education of their children (Goodall & Montgomery, 2014) with important consequences for student academic performance and wellbeing. Parental school engagement is expected to foster their own as well as their children’s satisfaction with the school. Similarly, previous studies showed that student school engagement predicts academic outcomes (Lei et al., 2018) and as such we expect that student engagement ultimately increases satisfaction with the school for the adolescents as well as their parents. We therefore hypothesized that:

**Hypothesis 2**: School engagement mediates the association between trust in the school and satisfaction with the school.

Taken separately the two core hypotheses are not surprising and were explored separately in previous studies. Our study aims to extend the insights on how trust, school engagement and satisfaction with the school are related in parent–adolescent dyads. We expect such an interplay to be relevant as close interpersonal interactions between parents and adolescents generate interdependence (Kenny, 1996, 2018) in the way they relate, in terms of attitudes and behavioral intentions, to the same target (school). We use the APIM approach (Kenny, 1996; Cook & Kenny, 2005) for testing mediation in dyadic data and add to the literature on school engagement by pointing to the relational dynamics that shape the interplay of trust, engagement and satisfaction with the school. APIM was recommended for studying close interpersonal relations such as family relations (Cook & Kenny, 2005) as it captures the interdependence in constructs evaluated in dyads engaged in close interpersonal interactions (Kenny, 2018).

## Method

### Sample and Procedure

Our study used a cross-sectional multi-source approach to collect data on trust, engagement and satisfaction with the school from parent-adolescent dyads. The study was part of a larger research project focused on attitudes toward schools in Romania and included high school participants in various regions of the country. Participants were invited to take part in the study via the school management team and were ensured of the voluntary nature of their participation as well as of the anonymity of their contributions. In order to match the parents with their respective child in this survey we have used anonymized codes that were generated using the same rules for the parent as well as for the child. We have matched the parent–adolescent pairs based on these anonymized codes. The final sample consisted of 506 parent–adolescent dyads for which we could accurately match the parents with their respective child. In the student sample 68.6% were females and the average age of the sample was 17.15 years old (ranging between 14 and 19), while in the parent sample 82.8% were female and the average age of the sample was 44.5 years old (ranging from 32 to 65). Age and gender of both adolescents and their parents were used as control variables.

### Scales

#### Trust in the School

For students, this was evaluated using three items by asking them to rate on a five-point scale (ranging from 1 - *Not at all* to 5 - *Very much*) their trust in colleagues, teachers and school in general. Our trust in the school scale for students evaluates trust in key interaction partners (colleagues and teachers) as well as trust in school in general, therefore it is aligned with the key tenet of SQT (Ward & Meyer, 2009) that trust emerges at the relational interfaces between the students and the school as the social system in which they are embedded. Cronbach’s alpha for this three-items scale was .86 showing a good internal reliability of the scale. Parental trust in the school was evaluated with a single item adapted from an overall indicator of trust as a generic evaluative tendency (Larson et al., 2018) “Overall to what extent do you trust your children’s school?” and recorded the answers on a five points scale ranging from 1 - *No trust at all* to 5 - *I trust it fully*.

#### Engagement With the School

School engagement was evaluated with different scales for the students and for parents. Typically, in the APIM models the same scales are used for both partners in a dyad, yet the engagement with the school differs significantly in terms of behaviors between parents and students, therefore we decided to use dedicated scales to measure parental and student engagement. Parental engagement was evaluated with five items presented in Schueler et al. (2017), example of items are, “How often do you meet in person with teachers at your child’s school?” and “In the past year, how often have you helped out at your child’s school?” (as in the original survey, answers were recorded on a 5-points scale ranging from 1- *Almost never* to 5 - *Weekly or more*). The scale is extensively used to assess parental school engagement (Margaritopoulou et al., 2024; Tucaliuc et al., 2024) and Cronbach’s alpha for this scale was .80 reflecting a good reliability of the scale. Student engagement was evaluated with five items adapted from Demerouti et al. (2010). The student engagement scale is in line with the Study Demands-Resources Theory (Bakker & Mostert, 2024) and evaluates students’ positive psychological states in relation to their activities at school, namely study dedication, absorption and vigor. Examples of items include “I always find new and interesting aspects in my study at school” (Dedication), “At school I am bored during classes” (item reversed coded for Vigor) and “I feel I am very absorbed in my school activities” (Absorption). Answers were recorded on a 5-points scale ranging from 1 - *Fully disagree* to 5 - *Fully agree* and Cronbach’s alpha for this scale was .78 reflecting a good reliability of the scale.

#### Satisfaction With the School

For students, this was evaluated by asking them to rate on a five point scale (1 - *Not satisfied at all* to 5 - *Very satisfied*) their satisfaction with the school, with the teachers and with the classes they attend. The satisfaction with the school builds on a bottom-up perspective on satisfaction (Ilies et al., 2019), and evaluates students’ satisfaction with the school as composite of satisfaction in relation to relevant activities and relational interfaces at school. As such, the scale includes the relational interfaces with teachers, as well as satisfaction with educational activities at school and with the school in general. Cronbach’s alpha for this scale was .86 reflecting a good reliability of the scale. Parental satisfaction with the school was evaluated with a single item adapted from a single item measure of job satisfaction presented in Wanous et al. (1997) phrased as “All things included how satisfied are you about your child’s school?”, answers were recorded on a five points scale ranging from 1 - *Not at all satisfied* to 5 - *Very satisfied*. Although single item measures are criticized for their psychometric properties, items that capture generic evaluative tendencies like satisfaction and trust provide good estimates of the overall underlying constructs (Allen et al., 2022; Nagy, 2002; Wanous et al., 1997).

## Results

We present the means, standard deviations and correlations among the variables in Table 1:

**Table 1:** Means, Standard Deviations and Correlations

Variable	Mean	SD	1	2	3	4	5	6	7	8	9
1. Gender parent	.17	.377	1								
2. Age parent	44.50	5.59	.155**	1							
3. Gender student	.31	.47	.171**	.006	1						
4. Age student	17.15	.70	-.008	.021	.047	1					
5. Trust parent	4.21	.86	-.002	-.036	.029	-.015	1				
6. Trust student	3.44	.85	.055	-.030	.063	.025	.329**	1			
7. Engagement parent	2.45	.85	-.036	-.074	.070	.024	.113*	.149**	1		
8. Engagement student	2.61	.68	.056	-.008	-.042	-.012	.281**	.630**	.196**	1	
9. Satisfaction parent	3.88	.98	.035	-.043	-.088*	-.012	.349**	.655**	.145**	.597**	1
10. Satisfaction student	4.15	.87	-.043	-.058	-.035	-.037	.616**	.316**	.148**	.294**	.388**

*Note.* Gender is dummy coded: Female = 0, Male = 1; dyadic correlations among variables are presented in bold.

*p < .05. **p < .01. ***p < .001.

For the multi-item scales used for students, we carried out a Confirmatory Factor Analysis and the measurement model with four distinct factors had a good fit with the data χ^2^ = 233.73, *df* = 41, *p* < .001, CFI = .93, TLI = .91, NFI = .91, RMSEA = .09, while the fit indices for the single factor model reflect a poor fit with the data χ^2^ = 449.74, *df* = 44, *p* < .001, CFI = .85, TLI = .82, NFI = .82, RMSEA = .13. In order to test our hypotheses, we first used OLS regression analyses with robust standard errors for the mediators and for the dependent variables. In the OLS analyses we have used the HC3 heteroskedasticity-consistent standard error estimator as recommended in Hayes and Cai (2007). The results of the OLS regression analyses are presented in Table 2:

**Table 2:** Regression Results for Mediators and Dependent Variables

Variable	Parental school engagement	Student school engagement	Parental school satisfaction	Student school satisfaction
Constant	1.76^†^ (.98)	1.03^†^ (.60)	1.94* (.78)	.89 (.76)
Gender parent	-.08 (.10)	.08 (.07)	-.08 (.078)	.03 (.07)
Age parent	-.01 (.01)	.002 (.004)	-.003 (.006)	-.003 (.005)
Gender student	.12 (.08)	-.13* (.05)	-.08 (.07)	-.14*(.06)
Age student	.02 (.05)	-.02(.03)	-.03 (.04)	-.01 (.04)
Parental school trust	.07 (.05)	.07* (.03)	.58***(.05)	.12**(.04)
Student school trust	.12*(.05)	.46***(.03)	.01 (.05)	.35*** (.04)
Parental school engagement			.06^†^ (.05)	.01 (.04)
Student school engagement			.14**(.05)	.56***(.05)
*N*	506	506	506	506
*R* ^2^	.04	.38	.39	.53
*F* statistic	3.04**	54.17***	29.41***	68.57***

*Note.* Unstandardized regression coefficients shown with robust standard errors between parentheses. Gender is dummy coded Male = 1, Female = 0.

^†^*p* < .10. *p < .05. **p < .01. ***p < .001.

As indicated in Table 2, parental trust has a positive and significant association with student engagement (β = .27, *p* = .01), while with parent engagement the association is positive as hypothesized in H1 yet it is not significant (β = .11, *p* = .18). Moreover, student trust in the school predicts significantly and positively both student engagement (β = .46, *p* < .001) as well as parent engagement (β = .14, *p* = .02). Based on these results we can conclude that Hypothesis 1 was only partially supported. An emergent result reported in Table 2 concerns the negative and significant association between student gender and student engagement with the school (β = -.09, *p* = .01) as well as a negative association between student gender and their satisfaction with the school (β = -.07, *p* = .03) showing that female students tend to report higher levels of engagement (*M* = 2.65, *SD* = .68) and satisfaction with the school (*M* = 3.79, *SD* = .83) than male students (for engagement *M* = 2.52, *SD* = .69 and for satisfaction with the school *M* = 3.60, *SD* = 1.00).

**Figure 1:**
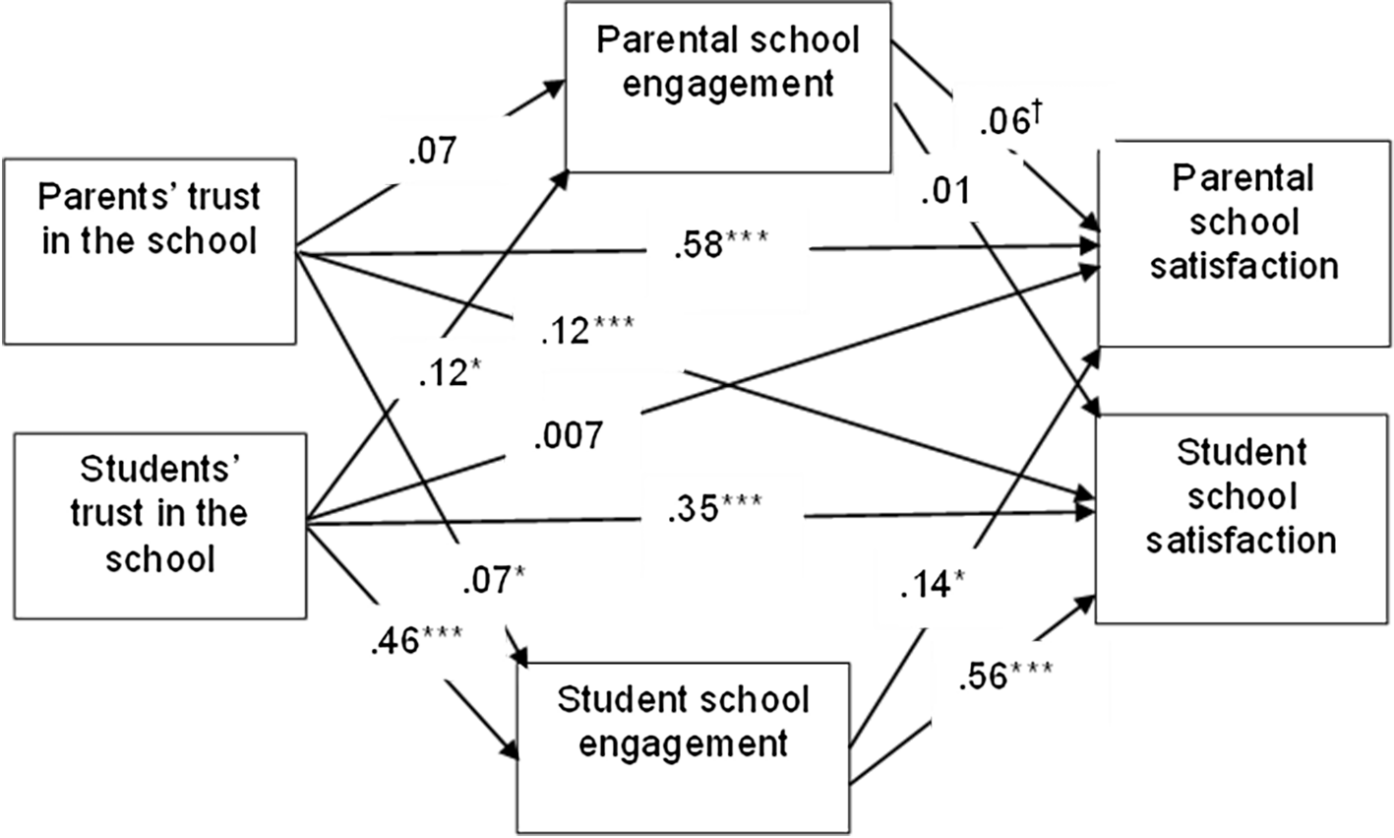
The Overall Results of the APIM Analyses *Note.* Unstandardized path coefficients are shown with robust standard errors between parentheses; Gender and age of parents and students were included as covariates in the model. ^†^*p* < .10. *p < .05. **p < .01. ***p < .001.

Our study collected data on trust, engagement and satisfaction with the school from dyads of parents and adolescents in line with the APIM methodology. One of the assumptions of the APIM analytic approach is that scores for the same variables are correlated within dyads, therefore in APIM, the level of analysis is the dyad and not the individual respondent (Cook & Kenny, 2005; Ledermann et al., 2011). As such our analyses are conducted on 506 parent-adolescent dyads with complete data on all variables included in the model and it estimates the mediating role of engagement with the school in the relation between trust in the school and satisfaction with the school (accounting for the covariance of student and parental evaluations in each of the dyadic measures). In line with these expectations, our results show that parental trust was positively correlated with the adolescent trust in the school (*r* = .33, *p* < .001) parental school engagement was positively correlated with adolescent’s engagement with the school (*r* = .20, *p* < .001) and finally parental school satisfaction was positively correlated with the adolescent’s satisfaction with the school (*r* = .39, *p* < .001). The mediating role of engagement in the relation between trust and satisfaction was tested using the MEDYAD procedure (Coutts et al., 2019). The MEDYAD procedure estimates the indirect effects of trust on satisfaction via engagement by taking account the correlations between the variables within dyads. We summarize the results of the MEDYAD analyses in Figure 1.

As illustrated in Figure 1 and summarized in Table 2 the mediation analyses reveal that the only significant mediator is student engagement with the school. The indirect effects are presented in Table 3 and all mediation paths via student engagement are significant, while the mediation paths via parent engagement are not significant as the confidence interval includes zero. Based on this pattern of results, we can conclude that student engagement is the only significant mediator in the relation between trust in the school and satisfaction with the school. This pattern of results puts the students at the center of the interplay between parental and student attitudes towards the school.

**Table 3:** Indirect Effects for All Paths Estimated in the APIM Mediation

Path	Indirect effect (SE)	95%CI
Parent Trust-->Parent Engagement--> Parent Satisfaction	.004 (.005)	[-.002;.01]
**Parent Trust-->Student Engagement --> Parent Satisfaction**	**.01 (.01)**	**[.001;.02]**
Parent Trust --> Parent Engagement --> Student Satisfaction	.001 (.003)	[-.006;.01]
**Parent Trust --> Student Engagement --> Student Satisfaction**	**.04 (.02)**	**[.01;.07]**
Student Trust-->Parent Engagement--> Parent Satisfaction	.01 (.01)	[-.002;.02]
**Student Trust-->Student Engagement --> Parent Satisfaction**	**.06 (.03)**	**[.01;.11]**
Student Trust --> Parent Engagement --> Student Satisfaction	.001 (.005)	[-.008;.01]
**Student Trust --> Student Engagement --> Student Satisfaction**	**.26 (.03)**	**[.20;.32]**

*Note.* Significant indirect associations are marked in bold

Given the fact that for students, trust in the school and satisfaction with the school were evaluated with multi-item scales, while for parents we have used single items to evaluate trust and satisfaction with the school, we have run the MEDYAD analyses using a corresponding single item for students as well (for students, we selected the items that matched the content of the items used in the parent surveys). As a robustness check we decided to run the APIM analyses using single item measures for trust in the school and satisfaction with the school for students in order to match the way these variables were assessed for parents. From the trust scale filled out by students, we have selected the question specifically referring to their trust in the school while from the student satisfaction scale we selected satisfaction with the school (the phrasing of these items were the same as the phrasing used for the parents to assess trust in school and satisfaction with the school). The APIM results using single item evaluations for students as well as parents (thus fully matched in terms of measurement approach) revealed the same pattern of significant indirect effects in which student engagement is the only significant mediator. The significant couple (dyadic) mediation via student engagement was fully supported in these additional MEDYAD analyses.

## Discussion

Our paper reports one of the first attempts to explore, using an APIM approach the interplay of trust, engagement and satisfaction with the school in parent–adolescent dyads. The most important aspect revealed by our analyses is that student engagement is the key mediator that explains the association between parental and adolescent trust in the school and parental and student satisfaction with the school. In the APIM terminology (Kenny, 2018) student engagement results from positive and significant actor and partner effects (a couple or dyadic effect), such that both parental trust as well as the students’ trust in the school significantly predict student engagement with the school, which in turn impacts on student and parental satisfaction with the school. In short, adolescent engagement in the school is a mediator in the couple (or dyadic) effect of trust on satisfaction with the school. Future studies on parental school related attitudes will benefit from including the dyadic interdependence of parent–children attitudes and behavioral intentions.

Moreover, students’ trust in the school is the only significant predictor of parental engagement with the school (in APIM terminology a partner only effect — Kenny, 2018), an important finding showing that future studies on antecedents of parental engagement with the school should extend their focus in order to include aspects related to children’s attitudes and behavioral intentions towards the school. We extend previous research on the role of student–teacher interactions as key antecedents of parental engagement with the school (Goodall & Montgomery, 2014) to show that parent–adolescent interactions are also relevant drivers of parental engagement with the school. These insights are also supported by the fact that when accounting for student trust, parental trust does not significantly impact on parental engagement with the school, therefore the relational dimensions rather than parental attitudes seem to be the key antecedents of parental engagement with the school. Previous empirical and conceptual analyses of parental school engagement chiefly focused on the relationship itself and considered classroom context, communication with the teachers and school management as key antecedents of parental engagement with the school (Fredricks et al., 2004; Goodall & Montgomery, 2014). In APIM terms, student engagement is dominating the mediation chain considered in our analyses, as parent engagement is explained by a partner only effect, while the student engagement is explained by a partner as well as by an actor effect (Kenny, 2018). According to our results, adolescents are really at the core of the relationship between their parents and the school, therefore future studies should build on the APIM approach to further explore parents’ relation with the school.

Our study also reveals a direct positive association between parental trust in the school and parental satisfaction with the school as well as the positive association between students’ trust in the school and their satisfaction with the school. These results are in line with the SQT (Ward & Meyer, 2009) that presents trust as a key antecedent of satisfaction with life in various social contexts. One of the tenets of the SQT is that trust is one of the key variables that connects the divide between individual and social systems as well as between individuals and institutional frameworks (Ward & Meyer, 2009). Our results support this view by pointing to school engagement as a mechanism that further explains how trust bridges this divide between parents and schools. An additional result reported in our regression analyses as well as the APIM approach is the direct association between parental trust in the school and adolescent satisfaction with the school. In other words, student engagement is only one of the possible mediators of the relation between parental trust in the school and student satisfaction with the school. This particular result shows the importance of using the APIM approach to test the interplay of attitudes towards the school in parent–adolescent dyads. We therefore call for more research that explores the way in which the parent–adolescent relations impact on the emergent school attitudes as well as other antecedents of engagement with the school.

A surprising aspect revealed by our APIM mediation analyses is the fact that when controlling for the covariance between student and parental trust in the school, the parental trust in the school did not significantly predict parental engagement with the school. In line with previous analyses that only looked at parental trust to engagement relationship (Houri et al., 2019), our bivariate analyses revealed a significant positive correlation between parental trust in the school and parental engagement with the school (*r* = .113, *p* = .01). Nevertheless, when taking into account students’ trust in the school (the only significant predictor of parental engagement, see Table 2), this positive relation between parental trust and engagement with the school becomes not-significant. This result once more, calls for more APIM studies on parent–adolescent dyads to explore parental engagement and satisfaction with the school. A particular area for future research concerns the role of dysfunctional cognition, in particular the role of distrust cognitive schema (Muntean et al., 2025; Tucaliuc et al., 2023; Tucaliuc et al., 2025), as antecedents of engagement and satisfaction with the school. Our paper addressed trust as a relational construct, yet personal dispositions as the tendency to engage in suspicious ruminative tendencies in relation to the school for example, can also be interdependent in parent-adolescent dyads. Future research could explore the joint influence of trust, as relational construct as well as personal disposition, on engagement and satisfaction with the school in parent-adolescent dyads.

Our results also revealed that male students tend to report lower levels of engagement and satisfaction with the school than female students. Such a result is in line with previous empirical results showing a higher engagement with school of female compared to male students (Mielityinen et al., 2023). Moreover, although in adolescence female students tend to report lower life satisfaction than male students (Marquez, 2024), our results show that female students report higher levels of satisfaction with the school, probably reflecting their engagement with the school and the fact that they tend to be less subjected to bullying and marginalization than male students are (Hosozawa et al., 2021; Muntean et al., 2024).

### Limitations

Next to its contributions, our study has several limitations as well. First, we have used single item measures for parental trust and satisfaction with the school. This raises two concerns, first the reliability concerns associated with the use of single item measures (Boyd et al., 2005) and second the difference in the way in which student and parental trust and satisfaction with the school were measured raises concerns for the APIM modeling. Previous studies have shown that single item measures capturing generic evaluative tendencies such as satisfaction and trust can be used when survey brevity is necessary to secure substantial participant response (Allen et al., 20222; Nagy, 2002; Tucaliuc et al., 2025; Wanous et al., 1997), as it was the case in our study. Second, in order to alleviate the concerns of different measures used in the APIM modeling, we have performed additional analyses with the corresponding items scales from the student survey that perfectly matched the single item measures used for the students and the results remained unchanged. A second limitation of our study was its cross-sectional nature. Although we have used multi-source data, we cannot draw definite causal claims concerning the associations between the variables included in the APIM mediation analyses. We did build the model based on the causal relations established in previous experimental research between parental trust and parental engagement (Houri et al., 2019) yet based on our APIM mediation analyses, we cannot draw definite causal claims (Michael & Hasida, 2024). Finally, the majority of our respondents, both for adolescents and parents were females and this can limit the generalization of our results. Women in general are more willing to participate in surveys (Curtin et al., 2000) and in the Romanian social context, in terms of parental engagement, they are more involved in children upbringing and education (Brumariu et al., 2020). Future research could attempt to replicate these results in different cultural contexts as gender differences and some additional cultural features could impact the results. In order to account for gender and other cultural differences in parental involvement, future studies could use samples from various cultures in which the parent-child dyads are balanced, so both parents fill in the survey.

### Practical Implications

Next to its academic contributions through the APIM approach, our results have also important implications for educational administrators. First, we build on the literature on trust as a key antecedent for engagement (Goodall & Montgomery, 2014; Houri et al., 2019) and we call for school administrators and managers to engage more in communicating with parents, create opportunities for feedback such that parental and student trust in the school is fostered. Trust is chiefly a relational construct and needs to be nourished in school–parent as well as school–student interactions in order to increase the level of engagement in school related activities. Second, in light of the dyadic effect reported in our study, we recommend focusing on parent–adolescent dyads in order to foster engagement with the school. Possible interventions could be centered around building learning communities in which students as well as parents are involved, therefore educational managers and school administrators could provide resources and platforms that make such learning communities possible. Previous research on fostering parental engagement already emphasized the need for a dual-navigation-approach that should focus on school as well as home parental involvement (Jeynes, 2018) in order to increase the quality and effectiveness of adolescent education. Finally, an emergent result of our study is that male students report less engagement and satisfaction with the school, therefore teachers, parents and school administrators should make sure that they facilitate and actively stimulate the engagement of male students, in order to also increase their satisfaction with the school.

### Conclusions

Our study uses an APIM approach to test the mediating role of engagement with the school in the relation between trust in the school and satisfaction with the school in parent–adolescent dyads. The results reveal a couple or dyadic (parent and child) significant mediation relations through student engagement in the school, such that students’ engagement in the school is positively and significantly predicted by both students and parental trust in the school and it positively and significantly predicts both student and parent satisfaction with the school. Moreover, our results reveal a partner only effect of student trust in the school on parental engagement with the school, opening fruitful venues for future research on parent–adolescent dyads. When student engagement was accounted for, parental engagement did not significantly play a mediating role in the relationship between trust and satisfaction with the school in the APIM mediation analyses, placing therefore student engagement as a core predictor of parental as well as student satisfaction with the school.

## Data Availability

The data analyzed in the current study are available from the corresponding author on reasonable and motivated request.
